# Self-assembly of hard anions around cationic gold nanorods: potential structures for SERS†

**DOI:** 10.1039/d4na00654b

**Published:** 2024-09-24

**Authors:** Offer Zeiri, Katherine M. Hatzis, Maurea Gomez, Emily A. Cook, Maegen Kincanon, Catherine J. Murphy

**Affiliations:** a Department of Chemistry, University of Illinois Urbana-Champaign Urbana Illinois 61801 USA murphycj@illinois.edu; b Department of Analytical Chemistry, Nuclear Research Center Negev P.O. Box 9001 Beer-Sheva Israel murphycj@illinois.edu

## Abstract

The placement of polyoxometalates next to the surface of noble metallic nanoparticles has been found to enhance the surface-enhanced Raman scattering (SERS) effect. The enhancement is believed to stem from either charge (electrostatic attraction) or chemical effects. Anisotropic gold nanorods are recognized as useful nanostructures for SERS, mainly due to the high electric field enhancement at their ends. The presented work examines the use of a polyoxometalate encapsulated gold nanorod for SERS, to assess whether the two enhancement pathways would be synergetic. For this, a gold nanorod-polyoxometalate composite was synthesized by coating cetyltrimethylammonium bromide-stabilized gold nanorods with a silicotungstic Keggin anion through electrostatic attraction. The structure was characterized, confirming that the nanorods have been fully encapsulated by the polyoxometalate. The SERS performance of the composite was assessed in solution using crystal violet as a SERS indicator, finding an analytical enhancement factor of 1.8 × 10^4^ in colloidal solution. The enhancement mechanism was examined first by comparison to gold nanorods stabilized by a cetyltriethylammonium bromide bilayer, cationic thiol bound polyoxometalate, and polyelectrolyte coating. Next, composites made using polyoxometalates of different atomic composition and charge were examined. It was concluded that the polyoxometalate charge had a noticeable effect on the enhancement while the atomic composition did not. Furthermore, high enhancement is observed mainly in cases where the nanorod monolayer allows the sequestration of the dye molecule into the nanoparticle's ligand layer. The proposed mechanism therefore involves the negative charge of the polyoxometalate attracting the positively charged dye, and facilitating the sequestration of the dye within the ligand bilayer, closer to the nanorod's surface.

## Abstract 2

A gold nanorod-polyoxometalte composite enhances SERS by combining electrostatic concentration of analyte and sequestration of it within the particle monolayer.

## Graphical abstract

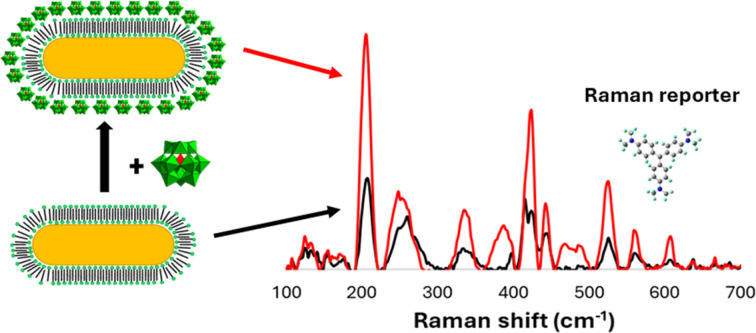

## Introduction

Gold nanoparticles represent a material state between bulk and atomic, exhibiting unique properties such as size- and shape-tunable optical and electrical properties, light-to-heat conversion, and high surface area, as well as chemical stability.^1,2^ Applications of gold nanoparticles include drug delivery,^3,4^ catalysis,^5^ photothermal therapy,^6^ medical imaging,^7^ electronics,^8^ and sensing.^9,10^ In the last field, one promising aspect of gold nanoparticles is their use in surface-enhanced Raman scattering spectroscopy (SERS).^11–13^ Generally speaking, Raman spectroscopy is an information rich but relatively insensitive technique. The presence of a noble metal particle (gold, silver or copper) next to a Raman active molecule can lead to incredible (up to 10^12^ under optimal conditions) enhancement of the molecule's Raman signal.^14^ Anisotropic nanoparticles are especially useful for SERS, as their shape allows for plasmonic tunability and hotspot generation.^13^ One of the most researched anisotropic nanoparticles are gold nanorods (GNR), which show great potential in SERS applications due to the enhanced local electric field they offer when illuminated with incident light, mainly at the GNR ends for longitudinal plasmon band excitation.^15–17^

Polyoxometalates (POM) are a diverse family of molecules, composed of early-transition metals bridged by oxygen atoms.^18,19^ Generally, POMs are easy to synthesize and characterize, stable, and inexpensive. These negatively charged inorganic clusters are versatile in their size, charge, electrical properties, and atomic composition. Consequently, they have found application in many fields, including catalysis,^20,21^ organics oxidation,^22,23^ energy storage,^24,25^ and sensing.^26,27^ POM have also found applications in nanoscience as metal reducers and nanoparticle stabilizing ligands^28^ and in hybrid materials.^29^ There have been several studies of the use of POM stabilized nanoparticles in SERS, mostly using silver nanoparticles,^30–33^ but recently some using POM stabilized gold nanoparticles have also been reported.^34–36^ The presence of POM next to the nanoparticle surface has been found to increase SERS signal. This effect has been attributed to either electrostatics, with the negatively charged POM attracting positively charged analytes, or to chemical enhancement.^29^ Streb *et al.*, using gold nanoparticles embedded in an iron-vanadate matrix, hypothesized that the enhanced signal originates from the electrostatic interaction between the positively-charged dye and the negatively-charged polyoxometalate, which increases the dye concentration near the SERS enabling structure.^36^ Gandía *et al.* reported enhancement using a polyoxometalate-decorated gold nanostructure, composed of either PW_12_O_40_^3−^ or PMo_12_O_40_^3−^.^34^ While both showed signal enhancement, using PW_12_O_40_^3−^ resulted in enhancement two orders of magnitude higher than using PMo_12_O_40_^3−^. This difference was attributed a chemical effect. DFT calculations found the HOMO–LUMO gap for the PW anion is larger than for the PMo anion (2.8 and 2.03 eV, respectively), resulting in better charge transfer from the gold core to the LUMO level of the POM, and from there to the LUMO level of the analyte (rhodamine R6G).

Despite the research involving POMs and nanoparticles, there are only a few examples in the literature of combining POMs and GNRs. Yang *et al.* have decorated GNRs with rings of POM, and used them to reduce silver on the GNR surface.^37^ The ring patterning is explained by the lower CTAB density at the GNR ends and electrostatic repulsion between POMs. Wang *et al.* combined the catalytic activity of the POM with the photothermal properties of GNRs, to produce a plasmon enhanced photothermal catalyst.^38^ A short cationic thiol was used to bind the POM close to the GNR surface, and photothermal conversion of a NIR laser irradiation used to increase local temperature near the POM, leading to enhanced catalysis. In the presented work, a GNR-CTAB-POM structure was assembled and characterized, revealing full POM coverage of the GNRs. The structure's potential for SERS was examined using crystal violet, and the enhancement mechanism explored.

## Experimental

### Instrumentation

Extinction measurements were performed on an Agilent Cary 5000 spectrophotometer, using a 1.0 cm quartz cell at room temperature. Purified water was used as the blank reference. Samples were diluted if necessary, after experiments established that sample dilution does not lead to changes in the absorbance spectrum for at least 10 minutes. Zeta potential measurement and dynamic light scattering (DLS) data were collected at 25 °C on a Malvern Zetasizer. Transmission electron microscopy (TEM) images were captured on a JEOL LaB6 2010 operating at 200 kV. Scanning transmission electron microscopy (STEM) and energy dispersive X-ray spectroscopy (EDS) elemental mapping was performed on a Thermofisher Scientific Talos F200x G2 STEM at 200 kV. Infrared spectra were measured using dried solutions on aluminum foil, using the attenuated total reflectance – Fourier transform infrared (ATR-FTIR) method on a Thermo Scientific Nicolet iS50 FT-IR. All spectra were obtained by averaging 32 scans at 4 cm^−1^ resolution over the spectral range of 4000–400 cm^−1^, and were processed using the Omnic software. ICP-OES measurements were performed on a PerkinElmer Optima 8300 after sample digestion. Raman measurements were performed with a B&WTek i-Raman plus system operating at 320 mW (at port) with a 785 nm laser. Integration of Raman peaks was performed using the BWSpec software.

### Chemicals

Aqueous solutions were prepared using deionized water purified by a Barnstead Nanopure II System (18 MΩ cm). Cetyltrimethylammonium bromide (CTAB, ≥99%), gold chloride trihydrate (HAuCl_4_·3H_2_O, ≥99.9% trace metals basis), sodium borohydride (NaBH_4_, >99%), sodium hydroxide (NaOH, ≥97%), silver nitrate (AgNO_3_, 99.9999%), p-hydroquinone (≥99%), crystal violet (1% solution in water), tungstosilicic acid hydrate (H_4_SiW_12_O_40_, purum p.a.), silicomolybdic acid (H_4_SiMo_12_O_40_, 21.2% solution), phosphotungstic acid (H_3_PW_12_O_40_, reagent grade), (11-Mercaptoundecyl)-*N*,*N*,*N*-trimethylammonium bromide (MUTAB), Poly(acrylic acid, sodium salt) solution (average MW ∼8000) were purchased from Millipore Sigma. Nitric acid and hydrochloric acid were purchased from Fisher Chemical. All chemicals were used as received. (16-Mercaptohexadecyl)-*N*,*N*,*N*-trimethylammonium bromide (MTAB) was synthesized according to published procedures.^39^

#### Gold nanorods synthesis

GNRs synthesis was based on an established seed-mediated method.^40^ All glassware was cleaned prior to use with freshly prepared aqua regia (HNO_3_ : HCl 1 : 3, volume : volume), washed thoroughly with deionized water, and dried. Seed and rod growth were kept at a temperature of 28 °C using a water bath. All stock solutions except the CTAB and HAuCl_4_·3H_2_O were freshly made. Any stock of HAuCl_4_·3H_2_O was kept protected from light.

#### CTAB coated gold seeds synthesis

9.5 mL of 0.1 M CTAB was added to a 20 mL scintillation vial equipped with a Teflon coated magnetic stir bar. Then, 0.5 mL of 0.01 M HAuCl_4_·3H_2_O was added and mixed evenly with rapid stirring. Next, 0.46 mL of 0.01 M solution of NaBH_4_ in 0.01 M NaOH, at ice-bath temperature, was added at once with rapid stirring to the gold solution. After a minute of stirring, the stir bar was removed, and the seeds were aged for 3 hours at a 28 °C water bath.

#### GNR synthesis

In a 1 L flask, 475 mL of 0.1 M CTAB was combined with 25 mL of 0.01 M HAuCl_4_·3H_2_O, and 0.7 mL of 0.1 M AgNO_3_ was added with stirring. Next, 25 mL of 0.1 M p-hydroquinone was added to the solution. 30 seconds after achieving a nearly colorless solution, 7.5 mL of the CTAB coated gold seeds was added all at once, and the flask was covered with parafilm and allowed to age undisturbed overnight at 28 °C. The next day the gold nanorods were centrifuged twice for purification (3000×*g* for 30 min). The pellets were diluted to 10 mL, with CTAB added to obtain a final CTAB concentration of 1 mM. The concentrated GNR suspension (*ca.* 6 nM) was kept in the dark. Nanorod dimensions (from ImageJ analysis of over 300 TEM images) were a length of 88 ± 9 nm, and width of 27 ± 3 nm (aspect ratio of 3.3). The nanorod extinction coefficient, obtained from the nanorod size and ICP-OES measurements, was found to be 3.2 (±0.2)–10^10^ M^−1^ cm^−1^.

#### GNR-CTAB-POM structure formation

A 10 mL solution of 0.1 nM GNRs was made by diluting a small volume of the concentrated GNRs solution with water and adding 0.1 mL of a 0.1 M CTAB solution. The solution was next centrifuged (2800×*g* for 13 minutes). The supernatant was discarded, and the pellet (∼0.2 mL) diluted with water to a volume of 10 mL. To increase the acidity of the solution prior to POM addition, 0.01 mL of 2% HCl were added to it, obtaining a pH of ∼3.5. To this solution a 0.2 mL volume of a 5 mM POM solution was added. The solution was then placed on a shaker for one hour. The structure remained stable for at least a month. Particles may settle at the tube bottom over time, but a short mixing redisperses them. The values provided here reflect samples containing 0.02 mM CTAB and 0.1 mM of POM. When different concentrations were required for specific experiments, volumes were adjusted accordingly.

#### Cationic thiol functionalization of GNRs

A 0.1 nM GNR solution was made by diluting the concentrated GNR solution with water. Next, a cationic thiol (either MUTAB or MTAB) solution was added to the GNRs, to obtain a final thiol concentration of 0.3 mM. The solution was placed on a shaker overnight. The next day the solution was purified by three rounds of centrifugation. Next, 2% HCl was added to the solution to obtain a pH of 3.5. Finally, 0.1 mM of POM was added to the solution, and it was placed on a shaker overnight.

#### Polyelectrolyte coating of GNRs

A poly(acrylic acid) solution was prepared by transferring 0.233 mL of a 8 k poly(acrylic acid) solution and 1 mL of NaCl (0.01 M) to a plastic tube, and diluting the solution to 10 mL with water. The tube was manually mixed by shaking before the next step. Next, 2 mL of the prepared PAA solution and 1 mL of a 0.01 M NaCl solution were added to a glass scintillation vial, 5 mL of a 0.15 nM GNRs solution added to the vial, and the solution was placed on a shaker overnight. The next day the solution was purified by centrifugation twice, and then diluted to give a final GNR concentration of 0.1 nM.

#### Raman measurements

Samples were prepared by mixing 1 : 1 volumes of a crystal violet solution (at the required concentration) and a 0.1 nM GNR-CTAB-POM solution in a 1.5 mL plastic Eppendorf tube. The sample was mixed using a vortex to ensure homogeneity and then sonicated for 1 min. Next, 0.05 mL of the sample was placed on a glass slide covered with aluminum foil. The drop was placed directly beneath the instrument's probe. The measurement time ranged from 1 to 10 s, depending on the intensity of the collected spectrum. Sample were measured 3 times, in a random order. A 10 s “dark” reading was obtained before measurements and subtracted from their spectra.

## Results and discussion

### Structure characterization

**Scheme 1:**
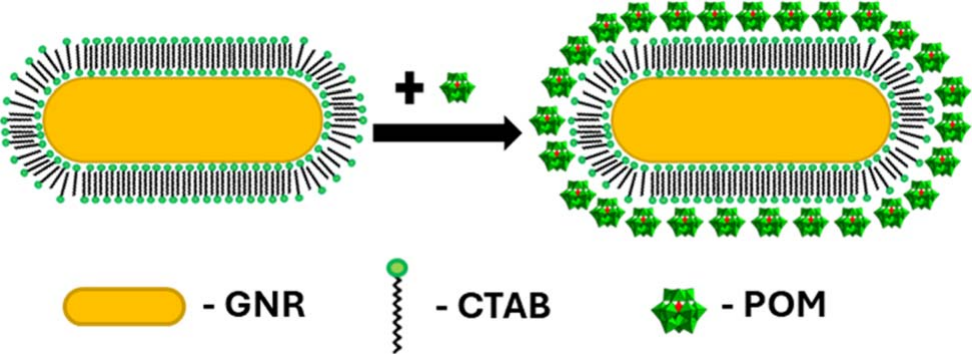
GNR-CTAB-POM structure. The cartoon represents a two-dimensional image of the structure; the CTAB bilayer and POM would cover all sides of the nanorod.

Seed-mediated gold nanorod synthesis, using silver and CTAB to break the symmetry of the seed growth, is one of the most common and well-developed gold nanorod synthesis methods.^41,42^ The nanorods produced using this method are stabilized by a CTAB bilayer, such that positively charged trimethyl ammonium headgroups are facing both the particle surface and the aqueous environment.^43^ Meanwhile, ionic bonding of negatively charged POM to cationic quaternary ammoniums is well documented.^44,45^ Therefore, addition of SiW_12_O_40_^4−^ (POM1) to GNR stabilized by a CTAB bilayer could be expected to produce a GNR-CTAB-POM1 structure, as shown in Scheme 1.

**Fig. 1:**
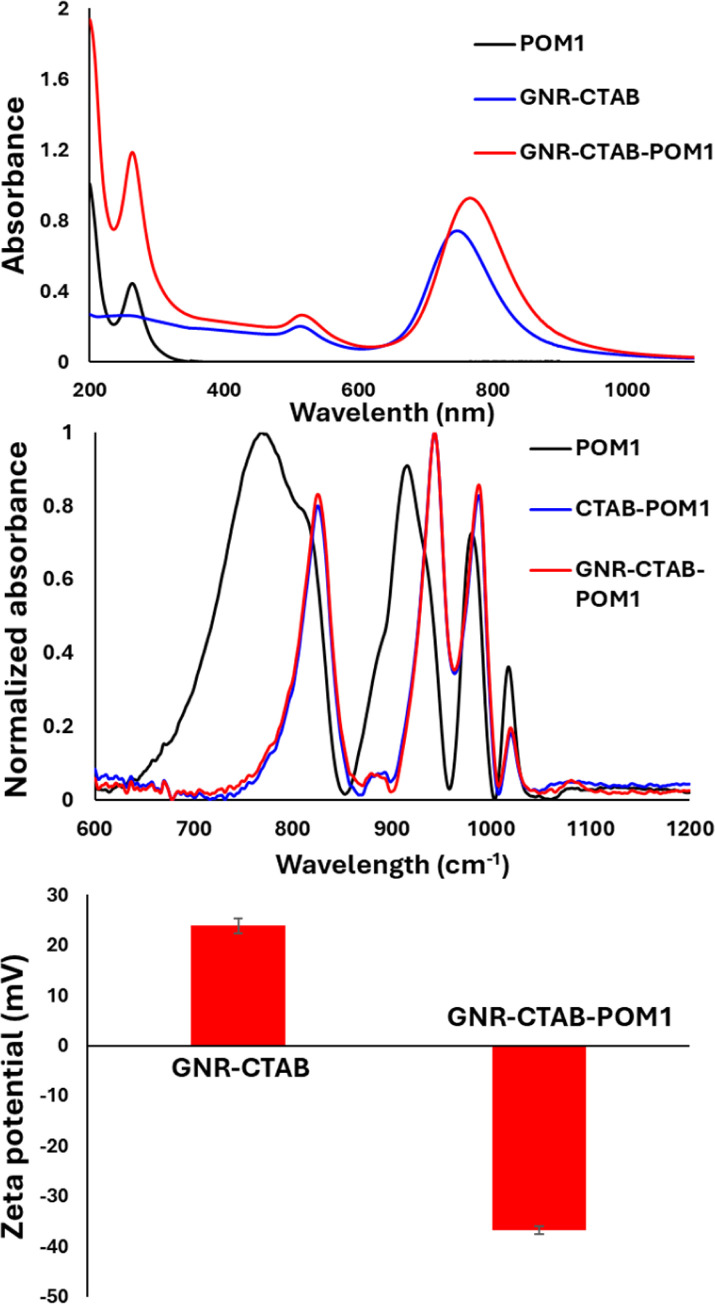
Top: UV-vis results for POM1 (0.1 mM), GNR-CTAB, and GNR-CTAB-POM1 (0.1 mM of POM1). After POM1 is added to the GNR-CTAB, a peak appears at 263 nm due to the presence of the POM, and the longitudinal and transverse plasmonic peaks red-shift and increase. Middle: FTIR results. The FTIR peaks red-shift when CTAB-POM1 complexes are formed. Peaks in the GNR-CTAB-POM1 structure are nearly identical to CTAB-POM1, supporting the formation of GNR coated by CTAB and POM1. Bottom: Zeta potential measurements. The surface charge of GNR is observed to change from positive to negative upon POM1 addition.

The binding of POM1 to the CTAB bilayer was verified using several methods: UV-vis and FTIR spectroscopy, zeta potential and DLS measurements, TEM imaging and EDS elemental mapping. UV-vis absorption measurement after addition of POM1 to the GNRs revealed a longitudinal SPR peak redshift (from 748 nm to 764 nm), and an absorbance increase (Fig. 1, top). This behavior is similar to that observed when POM monolayers are formed on gold nanospheres^46,47^ and originates from the large difference in refractive index between the inorganic POM and the organic stabilizing molecules. In addition, a peak appears at 263 nm, belonging to the POM1 molecule. The change in the SPR therefore suggests POM1 is present near the GNR surface. FTIR measurements were performed for solutions of POM1, a CTAB and POM1 mixture and the GNR-CTAB-POM1 structure (Fig. 1, middle). The addition of CTAB to POM1 slightly red shift some of the POM1 peaks due to the interaction formed between the CTAB and POM1. The GNR-CTAB-POM1 solution shows peaks nearly identical to those of the CTAB-POM1 mixture, supporting electrostatic interaction between POM1 and CTAB in the GNR solution, as well as the integrity of the POM1 molecule. Zeta potential measurements showed that the positive zeta potential of the CTAB-stabilized GNRs changes to negative after addition of POM1, further reinforcing the formation of the suggested structure (Fig. 1, bottom). While obtaining the precise hydrodynamic size for GNR by DLS is complex,^48^ the measurement results still provide information regarding the GNR size. Measurements of the GNRs before and after addition of POM1 (see SI, Fig. S1†) show the GNRs size increases after POM1 addition, supporting binding of POM1 to the CTAB bilayer.

**Fig. 2:**
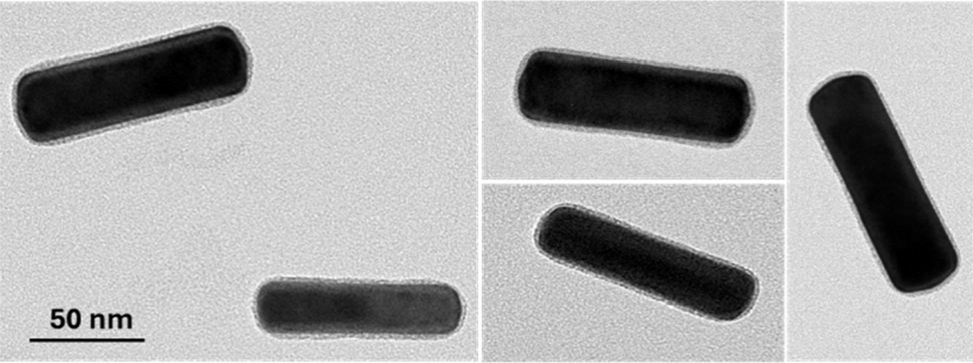
Transmission electron microscopy images of the GNR-CTAB-POM1 structure, revealing complete encapsulation of the GNR by the CTAB-POM1 monolayer. Additional images provided in the SI, Fig. S2.†

Next, a sample of GNR-CTAB-POM1 was imaged by TEM (Fig. 2, additional images provided in the SI, Fig. S2†). The concentration of POM1 in the sample was 0.02 mM (the lowest concentration for complete structure formation), as excess polyoxometalate hinder imaging upon drying. Due to the large electron density of the POM structures, they can be observed by TEM.^46,47^

The TEM images of the GNR-CTAB-POM1 show a 2.1 ± 0.5 nm (based on 220 measurements) layer of CTAB-POM1 surrounding the entire GNR. Considering the sizes of the CTAB bilayer and POM1 are *ca.* 3.2 and 1 nm, correspondingly,^46,49^ the size of the CTAB-POM1 layer observed is smaller than would be expected. While some structural changes in the bilayer could arise from binding POM1, these results have likely been affected by the sample drying prior to the TEM measurement. This is supported by the finding of images of GNRs featuring an asymmetrical, and sometimes significantly thicker (reaching over 7 nm) CTAB-POM1 layer (see SI, Fig. S3†), which we believe are the result of structure “deflation” during drying. It should be noted that this finding is in contrast to the findings of Yang *et al.*^37^ Their work took advantage of the differences between the CTAB ligand density on the longitudinal and transverse axes of GNRs (using GNRs with radii of 4.5–14.5 nm) to form POM rings only on the NRs longitudinal axis. In the work presented here, GNR with much thicker ends (27 ± 3 nm) were used, possessing higher CTAB densities.^50,51^ The higher CTAB density these GNRs have on their ends allows them to bind POM to the GNR ends as well as their sides. The POM coverage of the GNR ends could be vital for SERS, as the greatest enhancement of the electric field occurs at the GNR ends.^17^

**Fig. 3:**
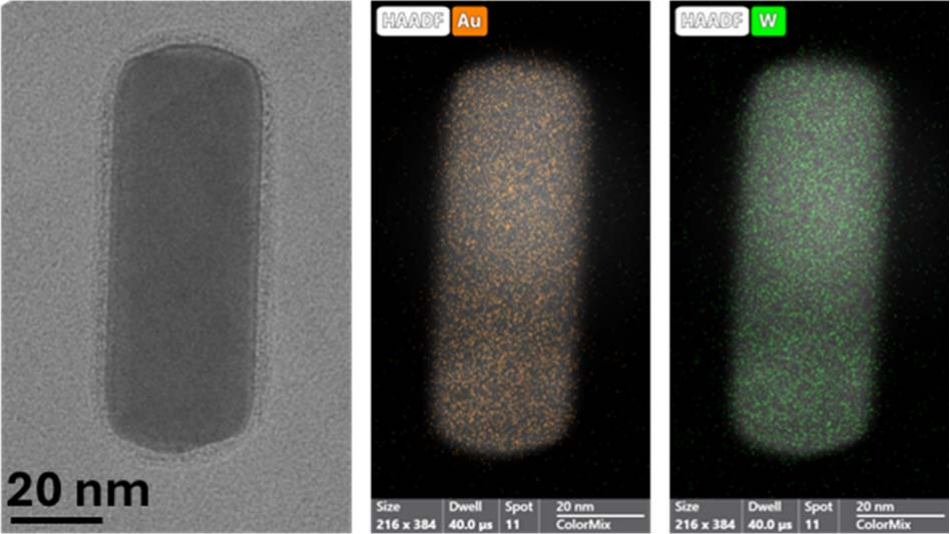
A TEM image (left) and elemental mapping of gold (middle) and tungsten (right) of the GNR-CTAB-POM1 structure, confirming the homogenous distribution of POM1 around the nanorod.

Finally, a sample of GNR-CTAB-POM1 was taken for EDS elemental mapping by STEM (after centrifugation to reduce excess POM). The distribution of gold and tungsten was measured (Fig. 3, additional images in SI, Fig. S4†), confirming that POM1 encapsulates the entire rod, with no clear pattern.

### Structure formation mechanism

**Fig. 4:**
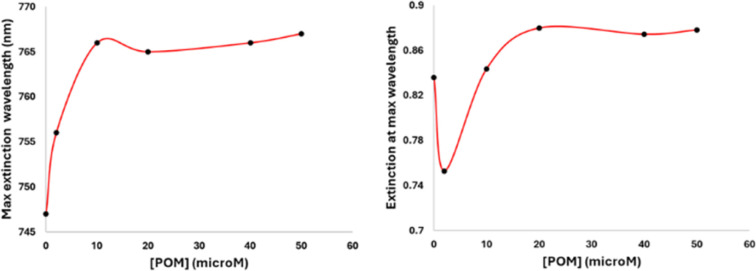
Monitoring changes in the maximum extinction wavelength (left) and extinction absorbance (right) of LSPR peak when POM1 (0, 2, 10, 20, 40, and 50 μM) is added to GNRs (final concentration of GNRs in all samples is 0.1 nM), measured 1 hour after POM1 addition. The maximum extinction wavelength increases and then plateaus as the system becomes saturated with POM1. The absorbance at the maximum extinction wavelength is observed to drop upon first addition of POM1 due to the formation of side-aggregates, rise as POM1 began to more fully coat the GNRs, and then reach a plateau point with excess POM1.

The formation process of the GNR-CTAB-POM1 structure was studied using the GNR extinction and zeta potential measurements after addition of increasing concentrations of POM1 (using GNR containing total CTAB concentration of 0.02 mM) (Fig. 4).

**Scheme 2:**
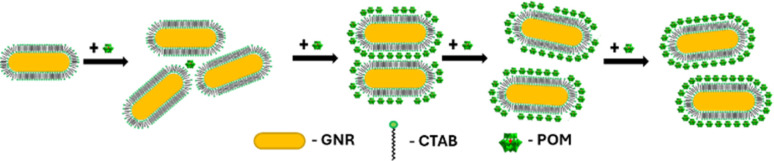
Formation of the GNR-CTAB-POM1 structure. At low POM1 concentration the POM1 leads to unstable aggregation of GNRs. Higher POM1 concentration leads to stable aggregates, then no GNR aggregation and finally full POM1 coverage of the GNRs.

As previously mentioned, the GNR-CTAB-POM1 maximum extinction peak red shifts relative to the CTAB stabilized GNRs. This shift was already observed at low POM1 concentrations (1, 2 μM), while the absorbance at the maximum peak decreased, indicating GNRs coming out of solution. As higher POM1 concentrations were added (5 μM) the maximum peak absorption began to increase, and the GNR longitudinal peak broadens, indicating formation of relatively stable aggregates. Aggregation was confirmed by DLS measurements (see SI, Fig. S1†). At higher POM1 concentrations aggregation was no longer observed, and the maximum peak absorbance kept increasing until *ca.* 20 μM of POM1 have been added. Addition of higher POM1 concentrations did not change the GNR spectrum further. Zeta potential measurements found that a 5 μM concentration of POM1 is required to turn the GNR zeta potential negative (see SI, Fig. S5†). Based on these results, the following formation process for the GNR-CTAB-POM1 structure is proposed (illustrated in Scheme 2). The aggregation observed at low concentrations of POM1 stems from CTAB on multiple GNRs binding to a single POM1 molecule. When POM1 concentration is very low (<5 μM), it provides a nucleation site for aggregation which leads to GNRs coming out of solution. At slightly higher POM1 concentrations (from *ca.* 5 μM) the GNRs are better stabilized by POM1, which is supported by zeta measurements turning negative. As a result, the aggregates formed are more stable, and remain in solution. At higher POM1 concentrations, the GNR coverage is high, and aggregation between GNR no longer takes place. The maximum peak absorbance increases until full coverage of the rods by POM1 is reached, after which further addition of POM1 no longer changes the number of POM1 molecules near the GNR surface.

**Fig. 5:**
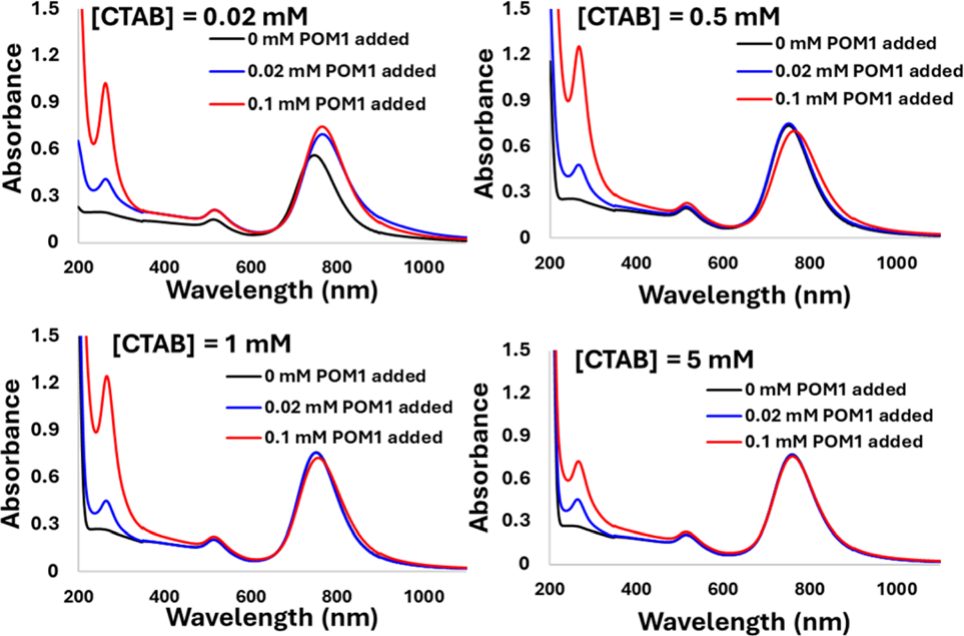
The addition of 0, 0.02 and 0.1 mM of POM1 to GNR-CTAB solutions containing total [CTAB] of 0.02, 0.5, 1 and 5 mM. As [CTAB] increases, the effect of POM1 addition on the SPR decreases.

The driving force for the GNR-CTAB-POM1 structure formation is presumably the ionic bond formed between POM1 and the quaternary ammonium of the CTAB. However, there are two types of CTAB in the GNR solution: “free” solvated CTAB, and CTAB organized in the GNR bilayer. To examine the interaction of POM1 with the two types of CTAB, GNR samples (0.1 nM) with total CTAB concentrations of 0.02, 0.5, 1 & 5 mM were prepared (free CTAB concentration, based on bromide ISE measurements, were 0.01, 0.48, 0.91, and 3.37 mM, respectively). Three concentrations of POM1 (0, 0.02 and 0.1 mM) were added to each of the samples (Fig. 5).

**Fig. 6:**
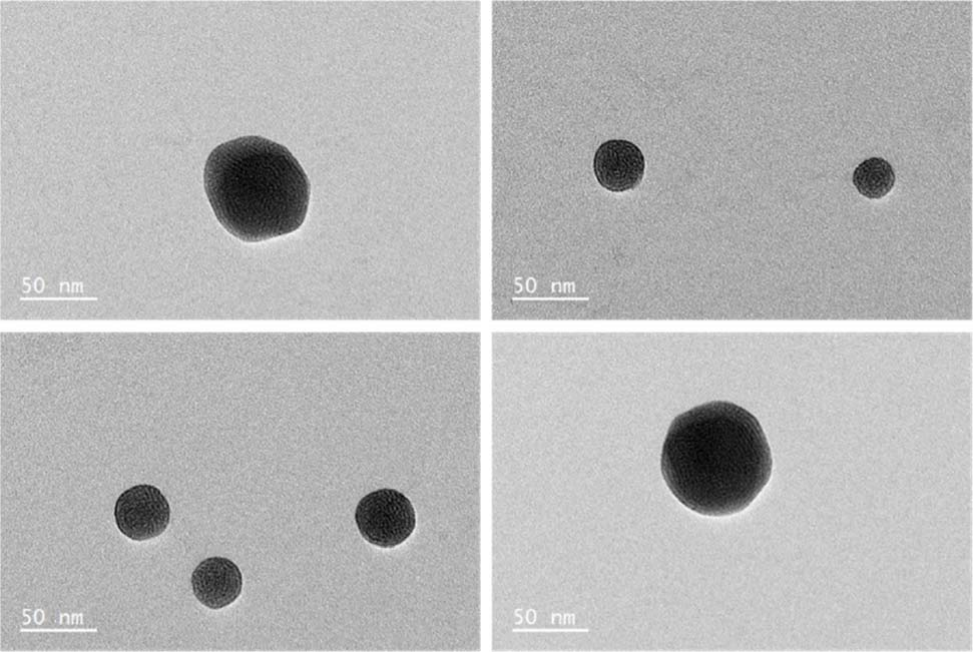
TEM images of a sample containing 0.05 mM CTAB and 0.1 mM POM1, revealing the formation of CTAB-POM1 vesicles.

In the case of 0.02 mM CTAB, which has the lowest free CTAB concentration, the formation of the GNR-CTAB-POM1 structure, observed by changes in the SPR and zeta potential, was observed for both POM1 concentrations. In the case of samples containing 0.5 or 1 mM of CTAB, the addition of 0.02 mM POM1 did not change the SPR or zeta potential of the rods. The addition of 0.1 mM of POM1 led to a small change in the SPR, which reflects the change observed when small POM1 concentrations are added to GNRs with low CTAB concentration. For the sample containing 5 mM of CTAB, neither 0.02 or 0.1 mM of POM1 caused any change in the SPR or zeta potential of the GNR. These results indicate that POM1 first reacts with the “free” CTAB, and only then binds to the CTAB in the GNR bilayer. Since the “free” CTAB concentration can vary between samples (due to small differences in centrifugations), an excess of POM1 (0.1 mM) was used for structure formation, with GNRs containing low (*ca.* 0.02 mM) CTAB concentrations. This result also raises the question of how do POM1 and “free” CTAB interact. Quaternary alkyl ammonium are known to form a variety of structures with different POM,^44,52^ and CTAB specifically has been shown to form both a lamellar-structure^53^ and micelle^54^ structures with POM. To determine the “free” CTAB and POM1 interaction, a sample of CTAB and POM1 (0.05 and 0.1 mM respectively) was prepared and examined by TEM, DLS and zeta potential. The TEM images reveal the CTAB and POM1 formed vesicles, with sizes ranging from 16 to 79 nm (Fig. 6).

According to the DLS and zeta potential measurements, the micelles have an average hydrodynamic radius of 109 ± 2 nm, and a zeta potential of −49 ± 2 mV. The difference between the results obtained by DLS and TEM could derive from sample polydispersity and from the sample drying prior to the TEM measurement. Preliminary electron microscopy data indicate that the observed vesicles are mutlilamellar, have a wall thickness of 1.4 ± 0.2 nm, and layer spacing of 3.1 ± 0.3 nm. Further study may uncover new nanoscale vesicle structures that could be of interest to numerous communities.

The stability of the structure is important for potential SERS applications.^13^ The GNR-CTAB-POM1 structure's stability was examined by two methods: stability to dilution and stability to centrifugation. A sample of GNR-CTAB-POM1 [0.1 nM] was measured at dilution factors of 4, 10, 25, 40 and 50. The maximum peak wavelength remained constant throughout all these dilutions, indicating the structure remains intact (POM1 is still located near the GNR surface). Next, a GNR-CTAB-POM1 sample was centrifuged multiple times, and its absorbance spectra measured after each centrifugation (see SI, Fig. S6†). During the first two centrifugations the only change observed in the absorbance spectra was a decrease in the POM1 peak at 263 nm, indicating excess POM1 was being removed from the sample by the centrifugation. It should be noted that the same GNR, without the addition of POM1, completely aggregate out of solution after the first round of centrifugation. Further rounds of centrifugation of the GNR-CTAB-POM1 structure led to a decrease in the spectra intensities of the GNRs, indicating some were aggregated out of the solution (aggregated particles could be observed at the bottom of some of the centrifugation tubes). However, the maximum absorbance peak remained constant throughout all of the centrifugation rounds. This result indicates that the POM1 molecules in the structure are in equilibrium with the “free” POM1 in the solution. The requirement of excess POM was also observed in POM stabilization of metallic nanospheres.^46^ The GNR-CTAB-POM1 structure is therefore robust to centrifugation, as long as excess POM1 is present in the solution. Samples of GNR-CTAB-POM1 were also analyzed using ICP-OES. The analysis found that there are 24 400 ± 900 molecules of POM1 for each nanorod, which remains constant throughout repeated centrifugations (details in SI, table S1†). This value represents the total number of POM in the samples, whether attached to the GNR or “free” in solution.

### Raman measurements

**Fig. 7:**
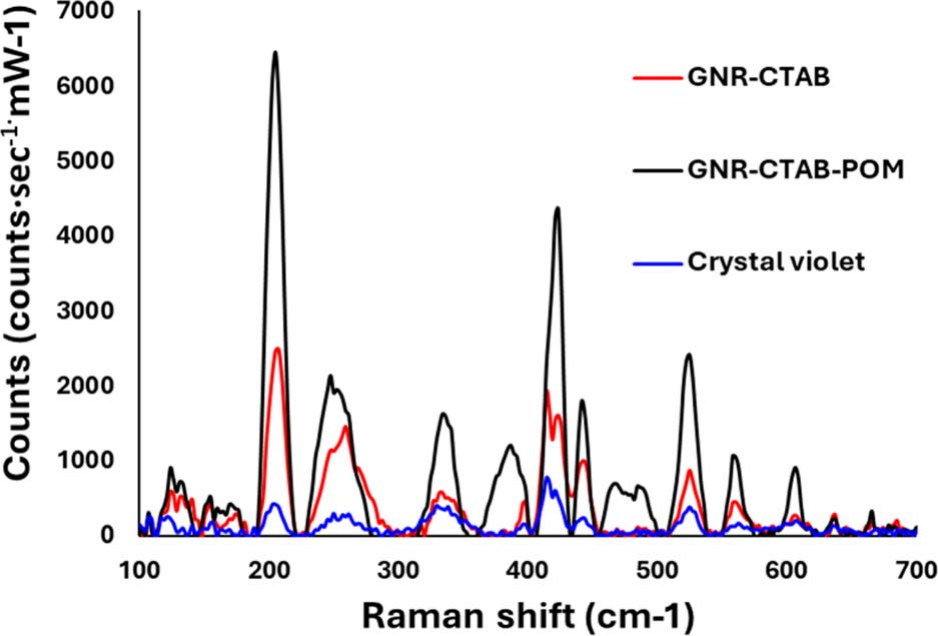
Spectra obtained for 0.5 mM of a crystal violet standard (blue), crystal violet with GNR-CTAB (black) and crystal violet with GNR-CTAB-POM1 (red). Both GNR samples are at a concentration of 0.1 nM.

Crystal violet was chosen as a model analyte to ascertain whether the GNR-CTAB-POM1 shows potential for SERS application. Raman spectra were collected for crystal violet without any GNR, with CTAB stabilized GNR (0.02 mM CTAB), and with the GNR-CTAB-POM1 structure (Fig. 7).

The presence of both GNR types (with and without POM1) leads to signal enhancement of the crystal violet Raman signal. However, the GNR-CTAB-POM1 enhancement was significantly higher than the CTAB stabilized GNR, indicating increased enhancement from the presence of POM1, similar to observations made using gold nanospheres.^34–36^ Solutions of POM1 and of a CTAB-POM1 mixture, without GNRs, showed no signal enhancement. The analytical enhancement factor (AEF),^14^ defined by eqn (1), is a quantitative value used to describe the signal enhancement a SERS system provides.
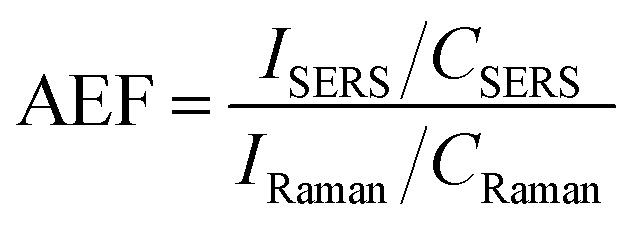
*I*_SERS_ – Raman signal intensity at a specific Raman shift when the SERS system is present, *I*_Raman_ – Raman signal intensity at a specific Raman shift when the SERS system is not present, *C*_SERS_ – the concentration of the Raman active molecule used for measurement with the SERS system, *C*_Raman_ – the concentration of the Raman active molecule used for measurement with the SERS system not present.

**Fig. 8:**
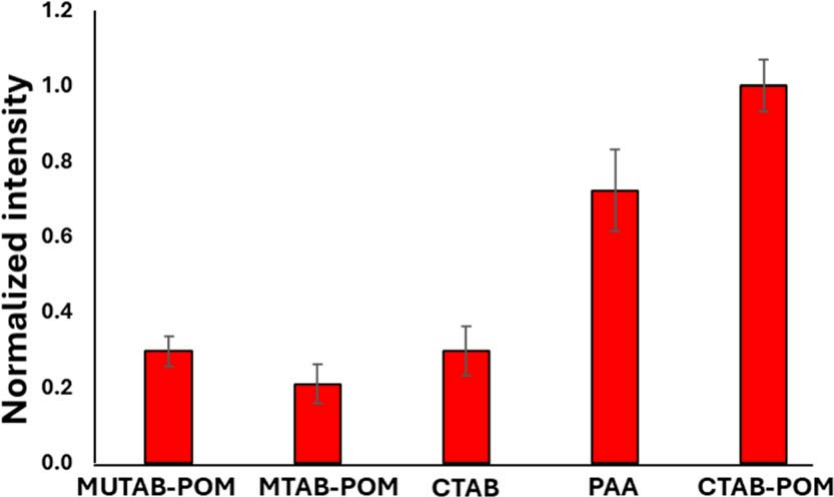
Raman signal intensity for crystal violet at 205 cm^−1^ obtained by differently functionalized GNR. The nanorod solutions used were all at a concentration of 0.1 nM.

The AEF for crystal violet measurements by the GNR-CTAB-POM1 structure was calculated to be 1.8 × 10^4^. Raman enhancement by POM is believed to be based on either on the POM charge (electrostatic attraction of the dye) or chemical enhancement,^29^ with some support for both. To better understand the enhancement mechanism, GNRs were functionalized by several manners. These include GNRs stabilized by CTAB, cationic thiols (MUTAB, MTAB), cationic thiols with POM1, and a polyelectrolyte coating. FTIR, zeta measurements and TEM images are available in the SI, fig. S7–S10.† Notably, complete coverage (including ends) by POM1 is also observed when the cationic thiols were used. Raman spectra of crystal violet (0.5 mM) using all the functionalized GNR types were collected and compared (Fig. 8).

**Scheme 3:**
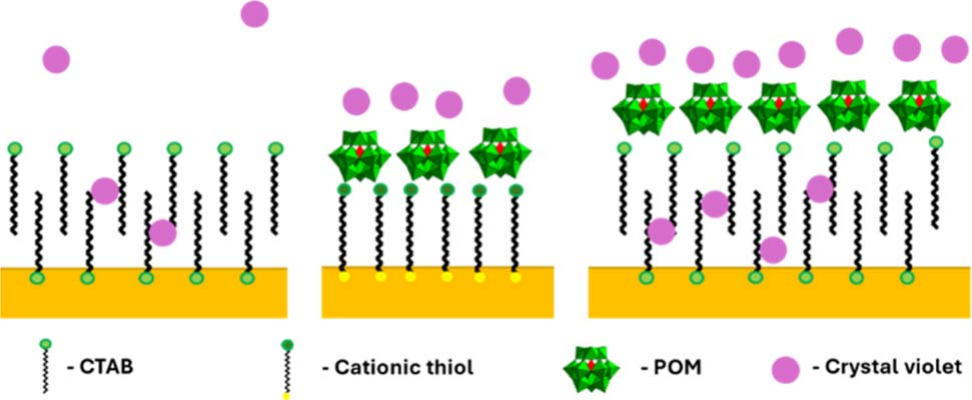
Suggested location of crystal violet molecules relative to the GNR surface. Left: GNR-CTAB, in which the dye can sequester into the CTAB bilayer. Middle: GNR-MUTAB-POM1 in which the dye molecules are attracted to the negatively charged POM. Right: GNR-CTAB-POM1 which both attracts dye electrostatically and sequesters it.

GNR functionalized by cationic thiols showed no enhancement at all, probably due to electrostatic repulsion between the positively charged thiols and the dye molecule. After binding negatively charged POM1 to the cationic thiols SERS is observed, with a higher signal obtained by the shorter of the thiols (lengths of 1.9 and 2.6 nm for MUTAB and MTAB, correspondingly. See SI, Fig. S11.† The difference in intensity was found significant by a *t*-test, *t* = 2.32, *t*_c_ = 2.78 for 95%). Since the cationic thiols are bound strongly to the gold surface, with headgroups densely packed and relatively immobile,^39^ the dye can be assumed to be located next to the negatively charged POM1. This indicates that the proximity of the dye to the GNR surface is important for signal enhancement. The enhancement obtained by the CTAB stabilized GNRs is similar to that of the short thiol with POM1. This might seem unintuitive, as the CTAB bilayer is longer than both thiols, and positively charged. However, it has been established the CTAB bilayer is capable of sequestering organic molecules,^55^ including organic dyes.^56,57^ So while not as many dye molecules are located next to the CTAB bilayer as for the cationic thiol-POM1 monolayer (similar to the cationic thiol stabilized GNRs, which show no SERS), some are sequestered into the bilayer. Those dye molecules are therefore closer to the gold surface on the GNR stabilized by CTAB then by those stabilized by thiol-POM1. The greater proximity of crystal violet to the surface seems to “make up” for the electrostatic repulsion of the dye by the positive charge of the CTAB bilayer. It can be hypothesized that using the GNR-CTAB-POM1 structure combines the electrostatic attraction by the negative charge of POM1, with the sequestering ability of the CTAB bilayer, resulting in improved enhancement of the Raman signal. The suggested dye location for each of the three GNR types is summarized in Scheme 3.

To test this hypothesis, CTAB stabilized GNRs were coated by PAA, and used for SERS with crystal violet (Fig. 8). Polyelectrolyte coated GNR also represent a negatively charged monolayer, which allows for dye diffusion to the nanorod surface.^58,59^ While the signal obtained by the PAA coated GNR is not as high as the GNR-CTAB-POM1, it is significantly larger than both the CTAB and MUTAB-POM1 coated rods. These results indicate that to obtain the highest signal enhancement, one requires an electrostatically attracting monolayer which can also sequester the organic dye into the monolayer of the nanorod. To further examine our hypothesis, the adsorption of crystal violet to GNR stabilized by MTAB-POM1 and CTAB-POM1 was quantified. For this, crystal violet concentrations were quantified from its absorbance, before and after exposure to the GNRs (GNRs were separated from the dye by centrifugation prior to measurement). This allows calculating the number of dye molecules taken up by each type of GNR. It was found that GNR-CTAB-POM1 adsorbs nearly twice as many dye molecules per nanorod as the GNR-MUTAB-POM1 (6800 ± 700 *vs.* 4100 ± 500). This method cannot differentiate between dye molecules electrostatically bound to the POM and those sequestered into the monolayer. However, the surface area of the two nanorods is similar, so it seems unlikely for the GNR-CTAB-POM1 to adsorb twice as many dye molecules as the GNR-MUTAB-POM1 unless they were being embedded into the nanorod ligand layer.

**Fig. 9:**
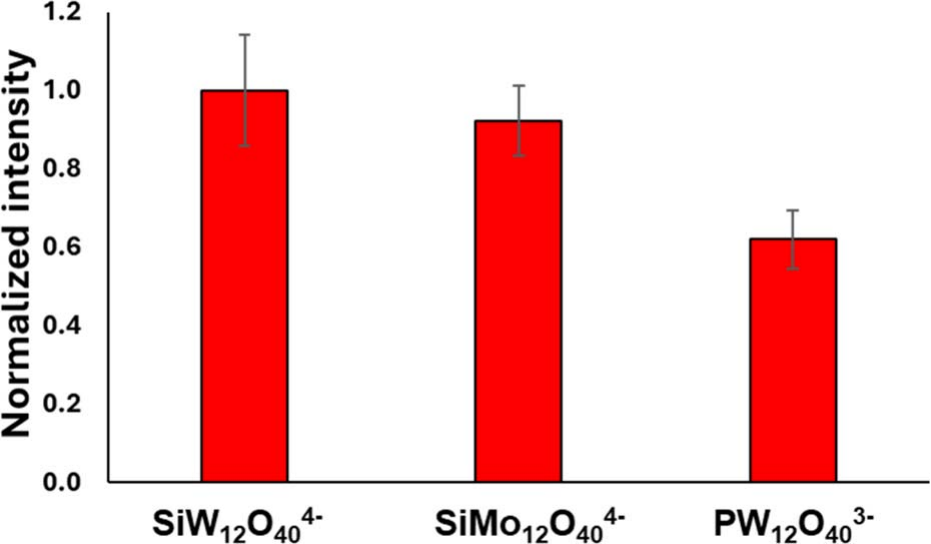
Raman signal of crystal violet at 205 cm^−1^ obtained by structures containing diff POM types. The nanorod solution used were all at a concentration of 0.1 nM.

These results suggest that in this structure the contribution of the POM to the signal enhancement is based mostly on the negative charge of the POM attracting the cationic dye, and not on a chemical enhancement effect between the dye and POM. Electrostatic attraction of analytes is known to play a significant role in SERS.^13,17,60^ To test this premise, GNR-CTAB-POM1 structures using 2 additional types of POM have been produced. The first employs SiMo_12_O_40_^4−^ (POM2) which shares the same charge as POM1 but is based on Mo oxygen bridges instead of tungsten. The second was PW_12_O_40_^3−^ (POM3), which is not as negatively charged as POM1. FTIR spectra ensured POM-CTAB binding and POM integrity for both POM, and zeta potential measurements and TEM images confirmed to same GNR-CTAB-POM structure (see SI, fig. S12–S15†). Raman measurements of crystal violet (0.5 mM) were obtained for all three structures (Fig. 9), and the signal intensities obtained were tested for significance in differences (using a *t*-test).^61^ Structures using the similarly charged POM1 and POM2 showed no significant difference (*t* = 0.82, *t*_c_ = 2.78 for 95%). On the other hand, GNR-CTAB-POM3 gave a significantly lower signal (*t* = 4.15, *t*_c_ = 2.78 for 95%). This result indicates the charge of the POM plays a significant role in the SERS enhancement of the GNR-CTAB-POM structure. It should be noted that these results do not rule out the option of some degree of chemical enhancement playing a role as well.

Based on all the results obtained in this work, further optimization of the GNR-CTAB-POM structure for SERS should focus on two design aspects. First, more negatively charged POM would likely improve the Raman signal enhancement, but care should be taken to ensure that repulsion between neighbouring POM will not interfere with the structure formation. Second, the GNR size should be optimized. Smaller GNR have a stronger “lightning rod” effect, and therefore increase SERS intensity.^62^ However, if the GNR ends are too small, and the CTAB density decreases, POM may not bind to them, diminishing the SERS intensity. Comparison to nanospheres stabilized by POM monolayers should also be undertaken to better understand the effect of the nanoparticle structure on the signal enhancement. Furthermore, as more GNR-POM composites are produced and studied, additional applications could be expected to emerge, such as in sensing,^26^ electronics^63^ and quantum materials,^64^ to take advantage of synergies between POM and GNRs.

## Conclusions

Gold nanorods, stabilized by a positively charged CTAB bilayer, were added to a solution with silicotungstic Keggin anions, forming a gold nanorod-polyoxometalate composite. Characterization of the structure confirmed the polyoxometalate was bound to the CTAB bilayer at the nanorod surface. The nanorod was found to be completely encapsulated by the polyoxometalate. Polyoxometalate located at the nanorod ends suggested the structure could be used for SERS, as the nanorod ends provide hotspots for electric field amplification, while polyoxometalates are known to enhance SERS when located next to metallic particles. Solution SERS measurements were undergone, using crystal violet as a model molecule. The composite displayed SERS activity, with an analytical enhancement factor of 1.8 × 10^4^ in colloidal solution. The enhancement mechanism of the structure was examined by comparison to measurements made using gold nanorods stabilized by a CTAB bilayer, cationic thiol bound polyoxometalate, and polyelectrolyte coating. The proposed mechanism suggests that the dye is electrostatically attracted to the composite by the POM while also being sequestered at the nanorod surface, and then experience the enhanced electromagnetic field of the GNR. While changing the composition of the POM did not have a significant effect on the Raman enhancement, using a lower charge POM decreased it, indicating the importance of the POM charge.

## Author contributions

Offer Zeiri – conceptualization, formal analysis, supervision, investigation, writing, methodology. Katherine M. Hatzis – software, methodology, writing. Maurea Gomez – formal analysis, investigation. Emily A. Cook – investigation. Maegen Kincanon – resources. Catherine J. Murphy – conceptualization, resources, supervision, funding acquisition, writing, methodology.

## Conflicts of interest

There are no conflicts to declare.

## Supplementary Material

NA-006-D4NA00654B-s001

## Data Availability

Raw data used in this work can be found in "Data for Self-Assembly of Hard Anions Around Cationic Gold Nanorods: Potential Structures for SERS. University of Illinois at Urbana-Champaign", https://doi.org/10.13012/B2IDB-8630796_V1.
