# Supplementary material for: Self-assembly of hard anions around cationic gold nanorods: potential structures for SERS
Source: Nanoscale Adv. 2024 Sep 24;6(24):6211–20. doi: 10.1039/d4na00654b (PMC11472117; doi:10.1039/d4na00654b)
Supplement: NA-006-D4NA00654B-s001 [file NA-006-D4NA00654B-s001.pdf]

Supplementary Information (SI) for Nanoscale Advances.
This journal is © The Royal Society of Chemistry 2024

Supporting information

Self-Assembly of Hard Anions Around Cationic Gold Nanorods: Potential Structures for SERS

Offer Zeiri[a,b]*, Katherine M. Hatzis[a], Maurea Gomez[a], Emily A. Cook[a], Maegen Kincanon[a], Catherine J. Murphy*[a]

a - Department of Chemistry, University of Illinois Urbana–Champaign, Urbana, Illinois 61801

b - Department of Analytical Chemistry, Nuclear Research Center Negev, Beer-Sheva, Israel, P.O Box 9001

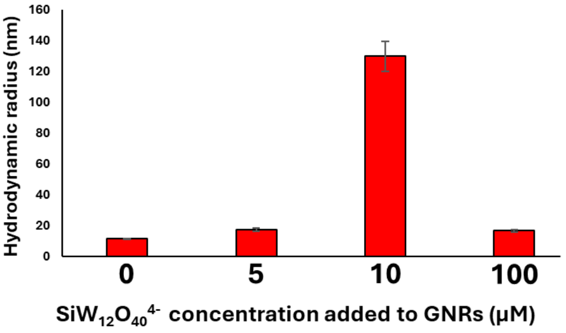

Figure S1. Hydrodynamic radii of GNRs after addition of different concentrations of **POM1**. At low concentrations of **POM1** aggregation occurs (exact concentration vary slightly between batches, due to variation in CTAB concentration). Once the GNR-CTAB-**POM1** structure is fully formed, aggregation is no longer observed. The final structure is found to be larger than the GNR-CTAB.

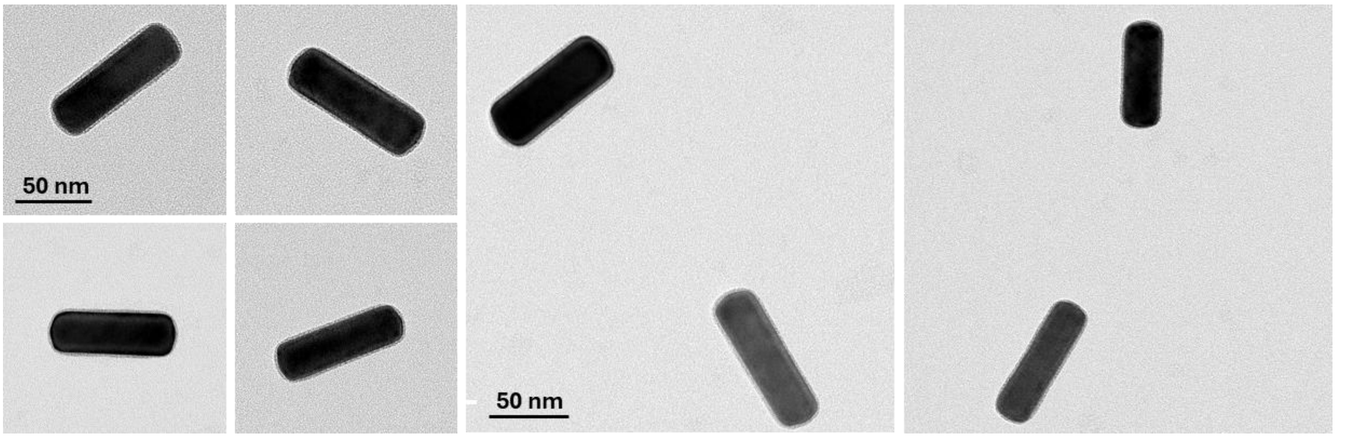

Figure S2. Additional TEM images of GNR-CTAB-**POM1**. Notice the scale bar is slightly different for the larger images to the right.

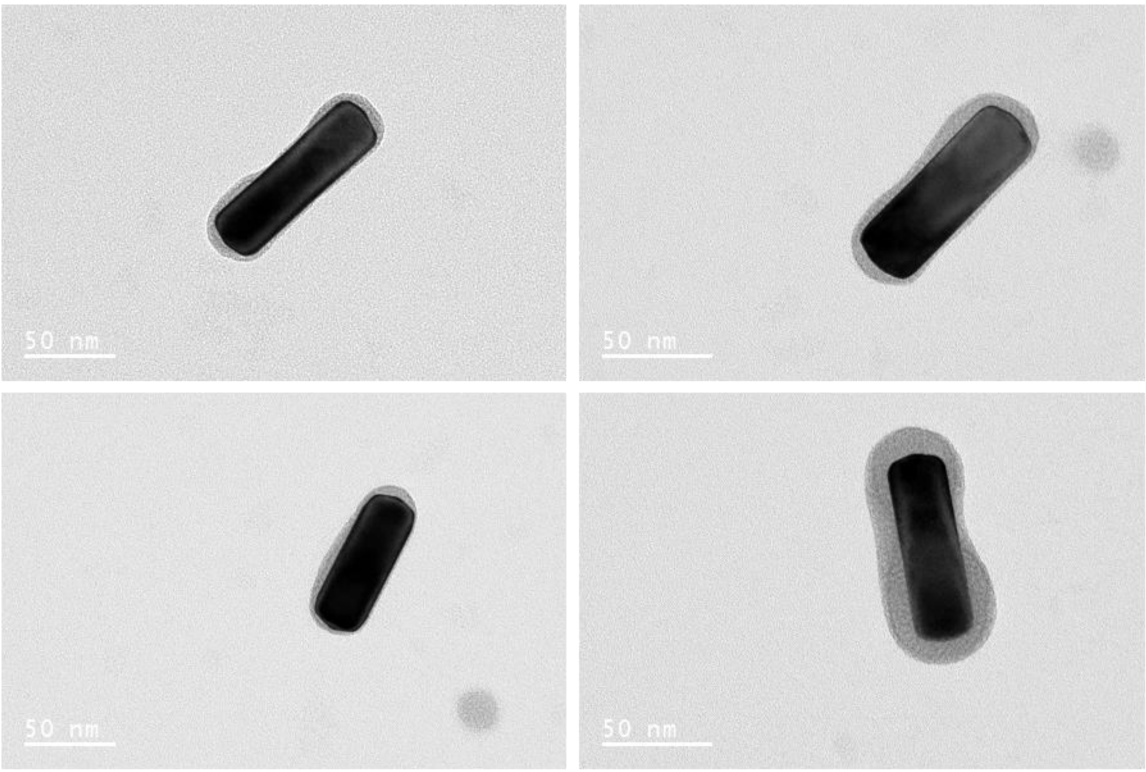

Figure S3. TEM images of GNR-CTAB-**POM1** which appear to have been "deflated" during sample drying.

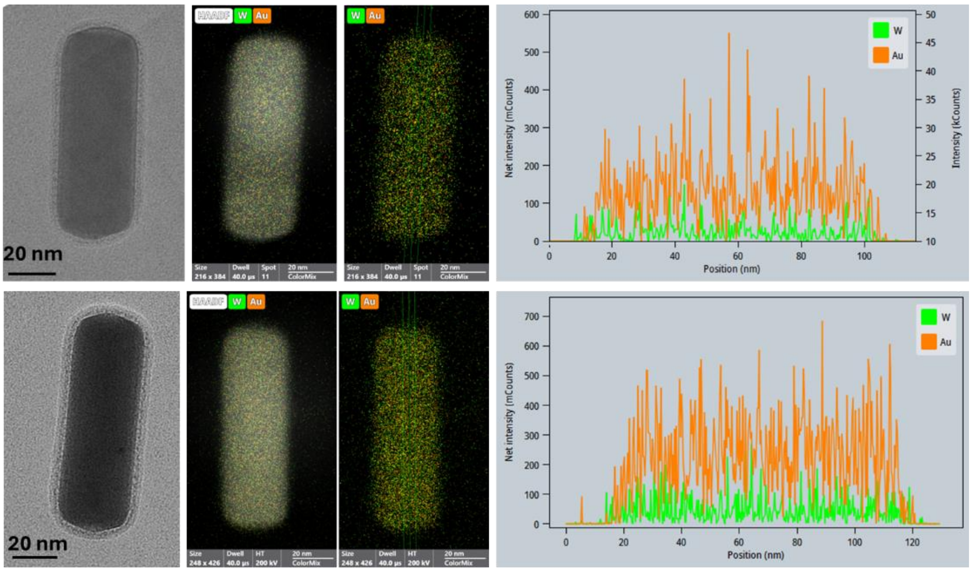

Figure S4. TEM image, gold and tungsten elemental mapping, and intensity profiles for two GNR-CTAB-**POM1** particles.

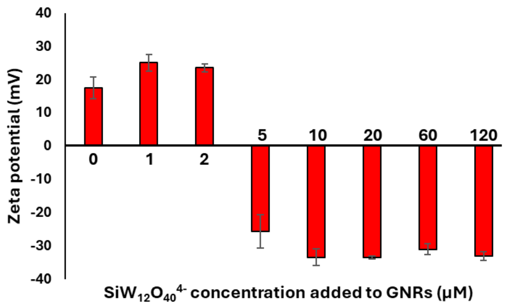

Figure S5. Zeta potential measurements of GNRs after addition of different concentrations of **POM1**. At low concentrations of **POM1** the GNRs are still mostly coated by CTAB, and remain positively charged. Once **POM1** concentration reaches ca. 5 μM (exact concentration vary slightly between samples, due to variation in CTAB concentration) the GNRs are coated by **POM1**, and therefore become negatively charged.

Centrifugation procedure and measurements

A 10 mL solution of GNR-CTAB-**POM1** was centrifuged (2800 xg, 13 min), forming a GNRs pellet. The supernatant was discarded (90% of the sample volume), and an equal amount of purified water added. The solution was shaken until the GNRs were well dispersed, and its absorption spectrum measured. The process was repeated four times (figure S5).

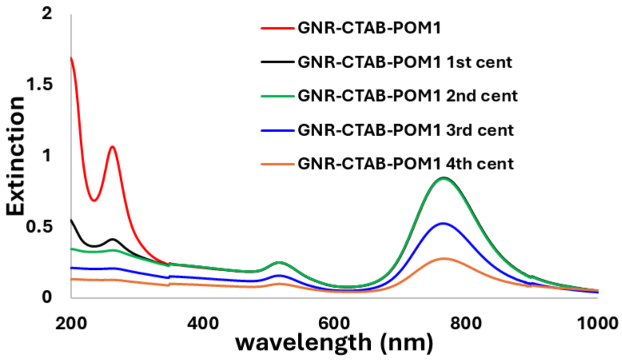

Figure S6. UV-vis extinction of GNR-CTAB-**POM1** after 0 to 4 rounds of centrifugation.

For the first two rounds of centrifugation the only change observed is the decrease of the **POM1** peak, due to removal of excess **POM1** from the solution. Further centrifugations show a decrease in GNR absorption due to GNRs aggregating out of solution. This indicates that the GNR-CTAB-**POM1** structure is stable to centrifugation as long as some amount of excess **POM1** molecules are present.

The concentrations of gold and tungsten in samples of GNR-CTAB-**POM1** after three and four rounds of centrifugation were measured by ICP-OES. Results are presented in table S1.

Table S1. Concentrations of gold and tungsten.

Number of centrifugations of the GNR-CTAB-POM1	[Au] mM	[W] mM	[GNR] nM	[POM1] mM	[POM1]/[GNR]
**3**	0.26	0.026	0.087	0.0022	25082
**4**	0.10	0.010	0.034	0.0008	23752

The ratio between **POM1** and the GNRs remained constant between the two samples, suggesting that 24417 ± 940 molecules of **POM1** per GNR are required for a stable structure. Note that this value represents a sample with practically no excess CTAB in the solution (after several rounds of centrifugation).

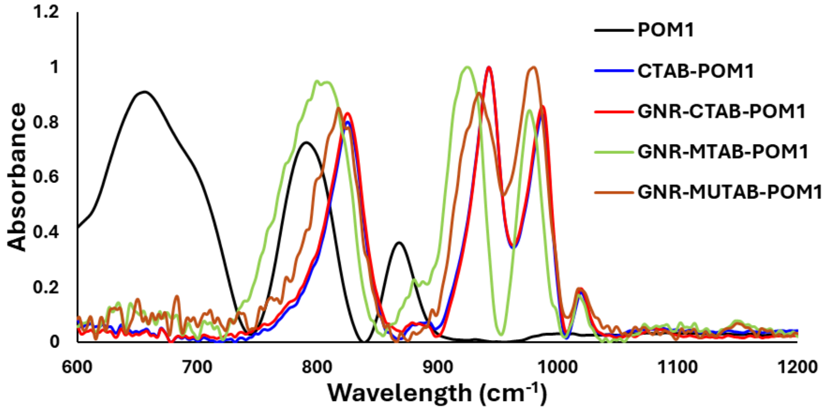

Figure S7. FTIR measurements of **POM1**, CTAB-**POM1**, GNR-CTAB-**POM1**, GNR-MUTAB-**POM1**, and GNR-MTAB-**POM1**.

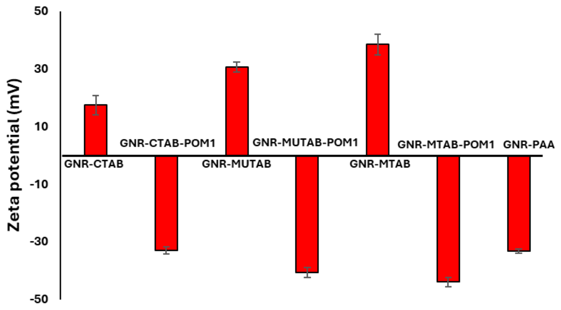

Figure S8. Zeta potential results for GNR-CTAB, GNR-CTAB-**POM1**, GNR-MUTAB, GNR-MUTAB-**POM1**, GNR-MTAB, and GNR-MTAB-**POM1**.

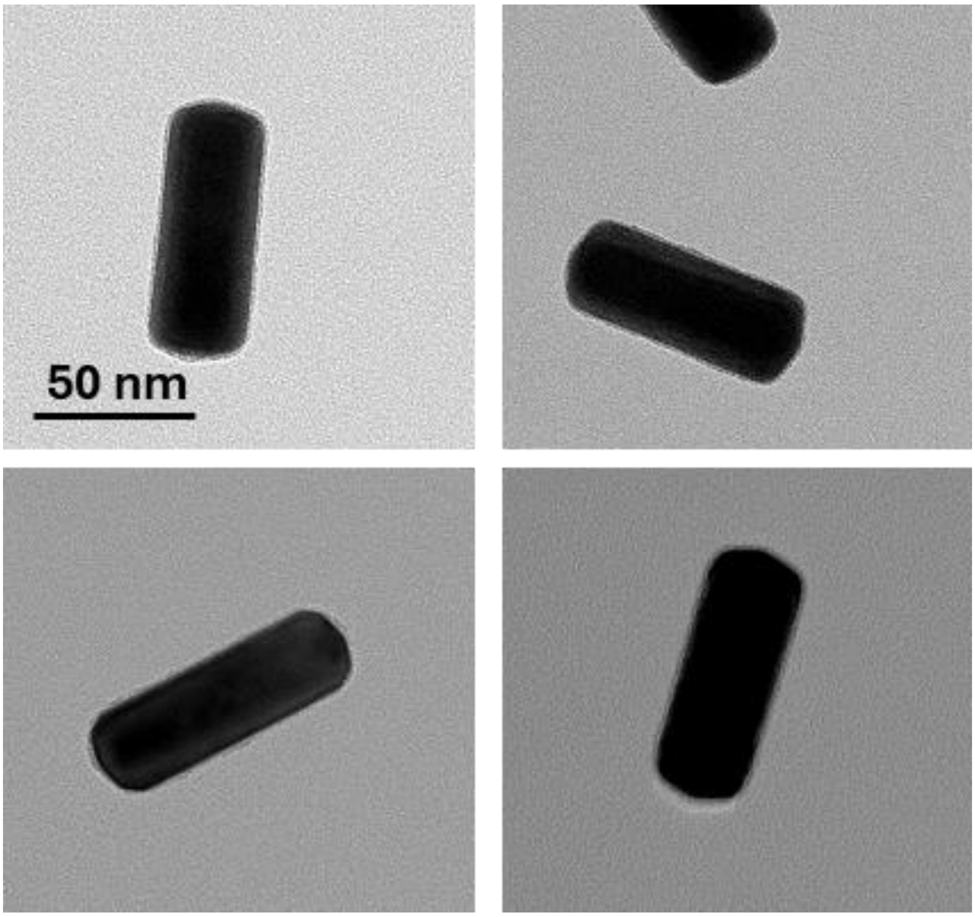

Figure S9. TEM images of GNR-MUTAB-**POM1**.

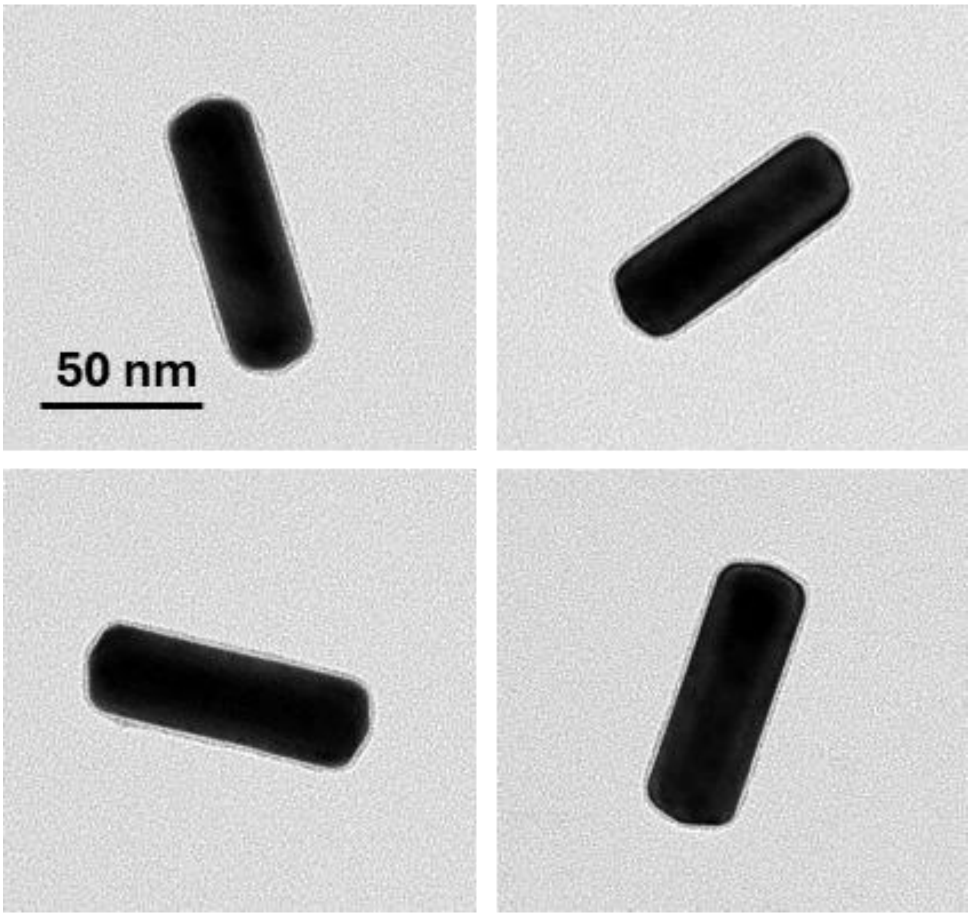

Figure S10. TEM images of GNR-MTAB-**POM1**.

Ligand length calculations

Geometry optimizations of ligand molecules for measurement were performed with density functional theory (DFT) calculations in the Gaussian 09 software.[1] These were conducted using the hybrid density functional B3LYP along with the def2-TZVP basis set to account for the electronics of the gold atom, and the solvation effect of water was taken into consideration via the solvation model density (SMD) model.[2]

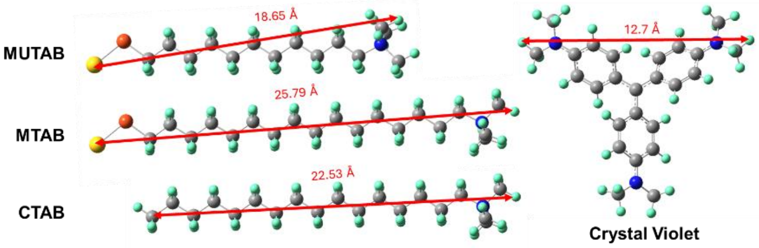

Figure S11. Left: Distance between gold surface to furthest hydrogen for MUTAB, MTAB and CTAB. Right: Distance between two periphery hydrogens of crystal violet.

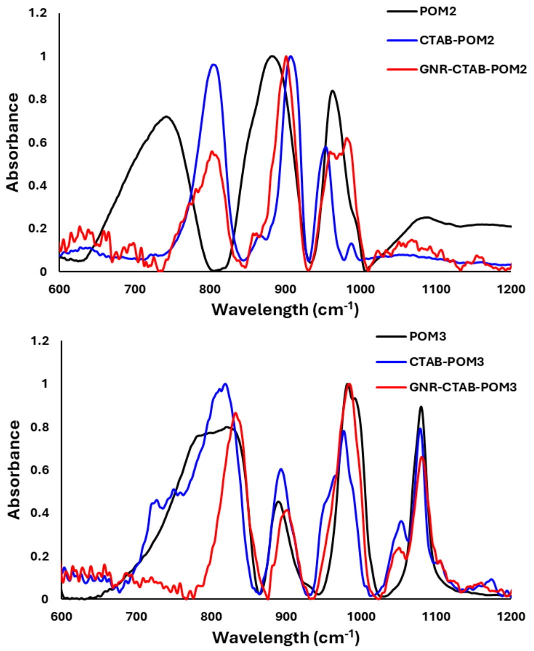

Figure S12. FTIR results for **POM2**, CTAB-**POM2**, GNR-CTAB-**POM2** (top) and **POM3**, CTAB-**POM3**, GNR-CTAB-**POM3** (bottom).

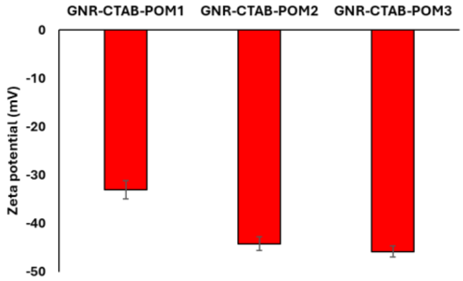

Figure S13. Zeta potential results for GNR-CTAB-**POM1,** GNR-CTAB-**POM2,** and GNR-CTAB-**POM3**.

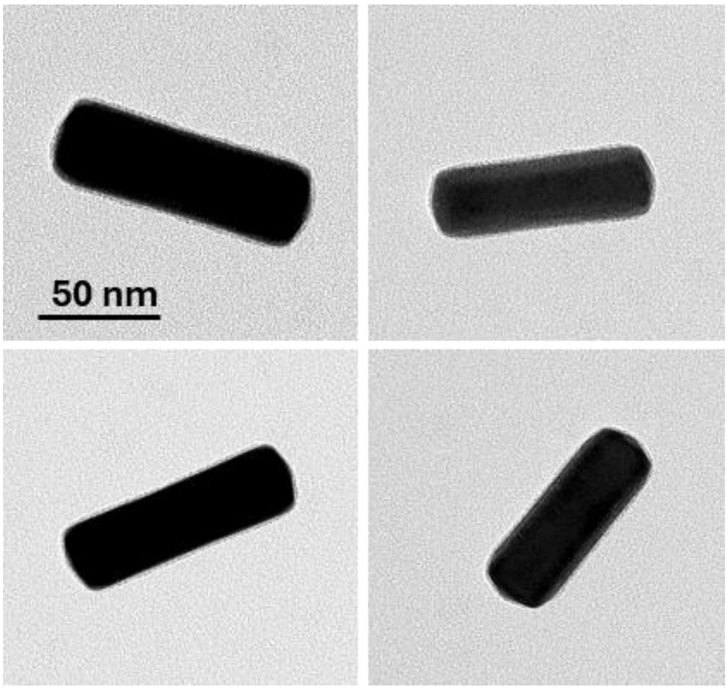

Figure S14. TEM images of GNR-CTAB-**POM2**.

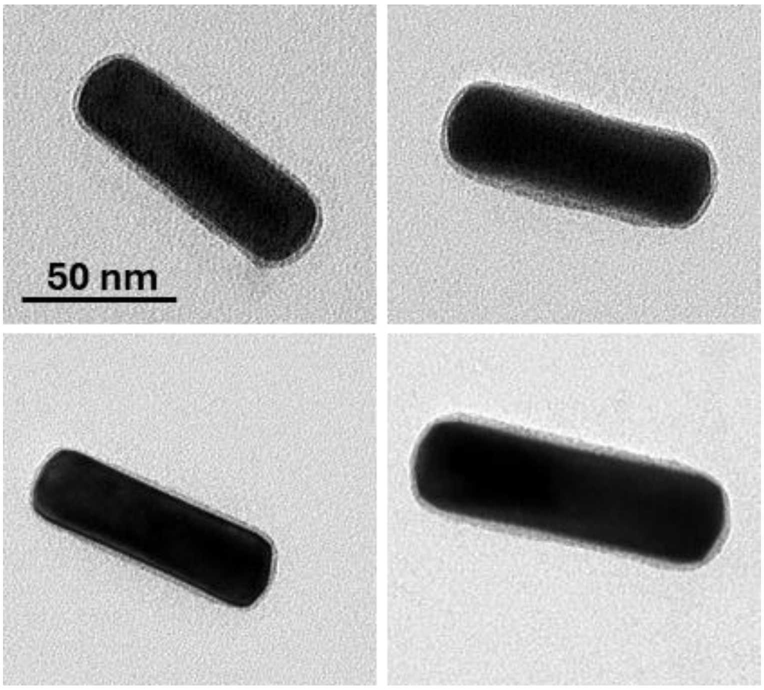

Figure S15. TEM images of GNR-CTAB-**POM3**.

(1) M. J. Frisch, G. W. Trucks, H. B. Schlegel, G. E. Scuseria, M. A. Robb, J. R. Cheeseman, G. Scalmani, V. Barone, G. A. Petersson, H. Nakatsuji, X. Li, M. Caricato, A. Marenich, J. Bloino, B. G. Janesko, R. Gomperts, B. Mennucci, H. P. Hratchian, J. V. Ortiz, A. F. Izmaylov, J. L. Sonnenberg, D. Williams-Young, F. Ding, F. Lipparini, F. Egidi, J. Goings, B. Peng, A. Petrone, T. Henderson, D. Ranasinghe, V. G. Zakrzewski, J. Gao, N.

Rega, G. Zheng, W. Liang, M. Hada, M. Ehara, K. Toyota, R. Fukuda, J. Hasegawa, M. Ishida, T. Nakajima, Y. Honda, O. Kitao, H. Nakai, T. Vreven, K. Throssell, J. A. Montgomery, Jr., J. E. Peralta, F. Ogliaro, M. Bearpark, J. J. Heyd, E. Brothers, K. N. Kudin, V. N. Staroverov, T. Keith, R. Kobayashi, J. Normand, K. Raghavachari, A. Rendell, J. C. Burant, S. S. Iyengar, J. Tomasi, M. Cossi, J. M. Millam, M. Klene, C. Adamo, R. Cammi, J. W. Ochterski, R. L. Martin, K. Morokuma, O. Farkas, J. B. Foresman, D. J. Fox. Gaussian 09, Revision A.02, 2016.

(2) Marenich, A. V.; Cramer, C. J.; Truhlar, D. G. *J. Phys. Chem. B* 2009, ***113*** (18), 6378–6396.
